# Micromagnetic and Quantitative Prediction of Hardness and Impact Energy in Martensitic Stainless Steels Using Mutual Information Parameter Screening and Random Forest Modeling Methods

**DOI:** 10.3390/ma18071685

**Published:** 2025-04-07

**Authors:** Changjie Xu, Haijiang Dong, Zhengxiang Yan, Liting Wang, Mengshuai Ning, Xiucheng Liu, Cunfu He

**Affiliations:** 1 College of Mechanical & Energy Engineering, Beijing University of Technology, Beijing 100124, China; zhanlu_yzx@163.com (Z.Y.); ningms@email.bjut.edu.cn (M.N.); 2 School of Emergency Equipment, North China Institute of Science and Technology, Langfang 065201, China; 3 China United Test & Certification Co., Ltd., Beijing 101407, China; dhaijiang2008@163.com; 4 School of Mechanical Engineering, Inner Mongolia University of Science and Technology, Baotou 014010, China; litingwang2021@imust.edu.cn; 5 School of Information Science and Technology, Beijing University of Technology, Beijing 100124, China; xiuchliu@bjut.edu.cn (X.L.); hecunfu@bjut.edu.cn (C.H.)

**Keywords:** micromagnetic testing, surface hardness, impact energy, random forests, mutual information, quantitative prediction

## Abstract

This study proposes a novel modelling approach that integrates mutual information (MI)-based parameter screening with random forest (RF) modelling to achieve an accurate quantitative prediction of surface hardness and impact energy in two martensitic stainless steels (1Cr13 and 2Cr13). Preliminary analyses indicated that the magnetic parameters derived from Barkhausen noise (MBN), and the incremental permeability (IP) measurements showed limited linear correlations with the target properties (surface hardness and impact energy). To address this challenge, an MI feature screening method has been developed to identify both the linear and non-linear parameter dependencies that are critical for predicting target mechanical properties. The selected features were then fed into an RF model, which outperformed traditional multiple linear regression in handling the complex, non-monotonic relationships between magnetic signatures and mechanical performance. A key advantage of the proposed MI-RF framework lies in its robustness to small sample sizes, where it achieved high prediction accuracy (e.g., R^2^ > 0.97 for hardness, and R^2^ > 0.86 for impact energy) using limited experimental data. By leveraging MI’s ability to capture multivariate dependencies and RF’s ensemble learning power, it effectively mitigates overfitting and improves generalisation. In addition to demonstrating a promising tool for the non-destructive evaluation of martensitic steels, this study also provides a transferable paradigm for the quantitative assessment of other mechanical properties by magnetic feature fusion.

## 1. Introduction

1Cr13- and 2Cr13-type martensitic stainless steels, after quenching and tempering, have good mechanical properties, corrosion resistance and impact toughness, so they are widely used in the manufacture of various key components such as turbine blades. Their impact energy and surface hardness are important for product quality control. The current evaluation of the mechanical properties of materials is mainly divided into two categories: destructive and non-destructive testing. The destructive hardness testing method quantifies the hardness value by indentation. Impact energy testing uses the Charpy impact test for destructive measurements. Non-destructive techniques include ultrasonic [1,2,3], acoustic emission [4,5] and micromagnetic testing (e.g., Barkhausen noise, incremental permeability, eddy current) [6,7]. Non-destructive testing methods other than micromagnetic testing characterise material homogeneity and integrity by capturing internal defects (e.g., cracks, inclusions). There are resolution limitations in resolving hardness and impact energy gradients. It is difficult to accurately map the spatial distribution of their mechanical properties. Micromagnetic detection technology is able to provide a high precision prediction of the mechanical properties (e.g., hardness) of complex microstructures (e.g., multiphase alloys). This is achieved by establishing a prediction model of the magnetic property parameters and mechanical properties through calibration experiments. Fraunhofer-IZFP in Germany developed a 3MA versatile micromagnetic testing instrument as an alternative tool for the non-destructive measurement of the mechanical properties and surface hardness of ferromagnetic materials [7,8,9].

MBN and IP are two typical methods of micromagnetic detection. Both types of micromagnetic signals are affected by microstructures such as magnetic domains, grain boundaries, dislocations and precipitates. The shape of the MBN butterfly curve is influenced by defect density and tissue composition so that its peak is positively correlated with the surface hardness [10,11]. When the domain wall motion is impeded, the MBN amplitude decreases with increasing hardness [12]. The MBN signal parameters (RMS and mean values, etc.) vary regularly with roughness due to the effects of nucleation and the extension of magnetic domain walls [13]. The MBN technique is applied to the evaluation of the embrittlement transition temperature of reactor pressure vessel steels [12]. The characteristic parameters in the IP curve have a correlation with the microstructure of creep specimen precipitates and recrystallization [14]. The yield strength of pipeline steels is influenced by average grain size and lattice friction [15]. By performing experimental calibrations on a limited number of specimens, it was possible to establish a quantitative relationship between the magnetic feature parameters (both MBN and IP) and surface hardness or impact work (determined using destructive measurement methods).

The accuracy of the prediction depends strongly on the prediction model and its correlation between the magnetic feature parameters and the target properties. A single magnetic parameter can visualize the correlation with mechanical properties. Many scholars have demonstrated the linear relationship between the MBN feature parameters (root mean square, peak and its position, etc.) and mechanical properties [10,16]. To achieve a simultaneous quantitative prediction of the various mechanical property indexes of different materials, multiple linear regression (MLR) models are constructed using the magnetic feature parameters of multiple types of magnetic signals. MLR models have been used to realize the on-line prediction of mechanical indexes such as strength and hardness [17]. In addition, some scholars have further improved the quantitative prediction of target properties by combining magnetic feature parameter screening with MLR modeling [18,19]. Intelligent prediction models, capable of applying complex mapping relationships, have been developed to make efficient use of magnetic parameters, which have a non-linear relationship with mechanical properties. Neural network models have been used to predict mechanical properties with better accuracy and stability than MLR models [20,21]. Generalized neural network models have been applied to obtain results showing that their prediction errors are relatively small [22,23]. The accuracy of the neural network prediction model for hardness predictions can be improved by optimizing the feature extraction technique for MBN and improving the repeatability of the instrument [24,25]. Pre-processing the data using Classification-Regression [26], or screening the parameters using Pearson’s correlation coefficient [27] and then combining them with neural network modeling predictions improves the accuracy of the predictions. In addition, other machine learning algorithms (e.g., support vector machines [28], random forests [29], genetic algorithms [7], etc.) have been combined with micromagnetic testing to improve detection accuracy [30]. Parameter screening methods, such as a Principal Component Analysis (PCA), are used to extract a subset of features from the high-dimensional micromagnetic signals that are strongly correlated with mechanical properties, and to establish an effective dimensionality reduction mapping, thereby improving prediction accuracy [28,30]. The above studies have shown that the combination of parameter screening and intelligent modeling techniques provides an accurate and non-destructive quantitative assessment of mechanical properties.

The above literature has mainly focused on exploring the correlation between certain individual magnetic feature parameters and hardness (or strength), but a strong linear relationship between magnetic parameters and impact energy has not been found. Therefore, it is not appropriate to use single parameter or MLR models to predict impact energy. The most common predictive models, neural networks, can fit complex non-linear relationships but rely on large amounts of labeled data and tend to overfit. The generalisation performance may degrade when the sample size is insufficient. Although Support Vector Machines perform better on small samples, selecting the kernel function and tuning the parameters depends on experience, and training time increases significantly with an increasing sample size. Random forest effectively captures the complex, non-linear relationships between variables by constructing multiple decision trees and using integrated learning. The generalisation of the model is enhanced by its mechanism of put-back sampling and feature random selection. A high level of accuracy can be maintained by averaging the predictions of several trees, particularly in scenarios with a small number of samples. Therefore, random forest is robust to non-linearity, overfitting resistance and small data.

This study innovatively proposes a quantitative prediction method combining mutual information value feature screening and random forest modeling for martensitic stainless steels (specifically, 1Cr12 and 1Cr13). The method aims to overcome the challenges of the complex mapping relationships between magnetic feature parameters and target properties, as well as the relatively limited amount of data. A three-step strategy is followed in order to achieve a high precision prediction. First, the instrument configuration is optimised to ensure the fidelity and resolution of the raw signal acquisition. Then, a subset of high-dimensional micromagnetic features that are strongly correlated with the mechanical properties (hardness and impact work) are extracted using mutual information value feature parameter screening to establish an effective dimensionality reduction mapping. Finally, a random forest model is used for global optimisation to ensure that the predictive model generalises well.

The organization of the subsequent parts of this paper is outlined below. Section 2 (Experimental) describes the sample preparation procedure, the hardness and impact energy measurements, and the composition of the micromagnetic test system and its basic principles. Section 3 (Feature Parameter Screening) explores the equations for applying mutual information values in feature parameter screening and their effects. Section 4 (Results and Discussion) demonstrates the construction steps of the random forest model and its predictive results. Finally, the conclusions of the whole paper are presented in Section 5.

## 2. Experimental

### 2.1. Specimen Preparation

**Figure 1:**
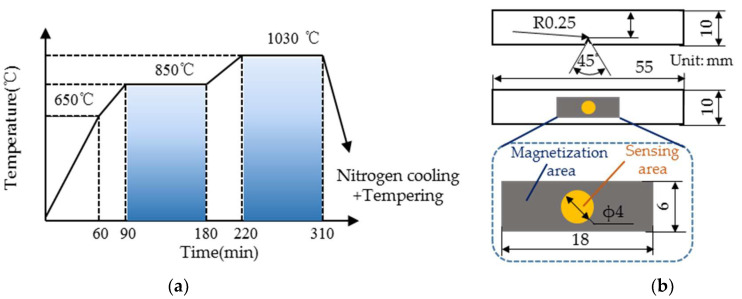
(**a**) Heat treatment process and (**b**) Dimensions of impact specimens.

In this study, two martensitic stainless steels (1Cr13 and 2Cr13) were selected to prepare the heat treatment specimens. Chemical composition is shown in Table 1. Both materials were subjected to the quenching and tempering processes. During the quenching process, all the heat treatment specimens were gradually heated up to 1030 °C, followed by nitrogen cooling to room temperature. Temperature and holding time are shown in Figure 1a. The quenched specimens were tempered successively for 240 min and divided into different batches. The hardness and impact energy of the tempered specimens were adjusted by changing the tempering temperature (in the range of 200 to 800 °C). After heat treatment, the specimens were subjected to wire cutting and surface polishing to prepare the standard hardness and impact specimens. The 13 and 14 hardness specimens were prepared for 1Cr13 and 2Cr13, respectively. The standard impact specimens of 1Cr13 and 2Cr13 were divided into 13 and 14 batches (3 specimens per batch according to the national standard [31]), respectively, and are sketched in Figure 1b.

**Figure 2:**
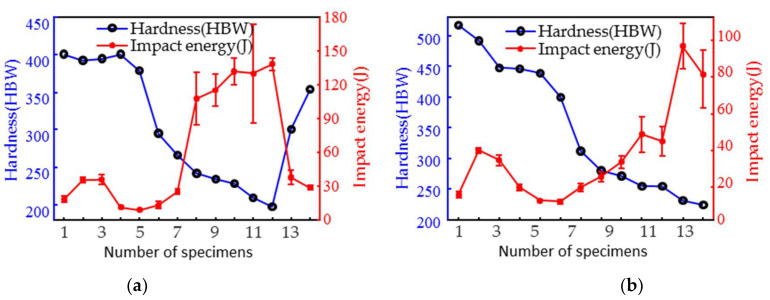
(**a**) 1Cr13 and (**b**) 2Cr13 hardness and impact energy.

To achieve a non-destructive quantitative evaluation of the surface hardness and impact energy for the two materials, it is necessary to acquire magnetic signals and measure the mechanical properties as calibration data. The MBN and IP signals of the two materials (all specimens) were first tested with a micromagnetic testing system, and then a micro-hardness tester was used to test the surface Brinell hardness value. It is worth noting that the micromagnetic testing and hardness measurements were carried out in the same area. To ensure the accuracy and reliability of the test results, three tests were conducted at close positions on the specimen, and the average value was calculated. The hardness test data for both materials showed a high degree of stability. Standard deviations were controlled to within 5 HBW and the coefficients of variation were less than 3%. This low variation result is an indication of the very high consistency of the material hardness values across the test conditions. The robustness of the material hardness index is further confirmed by the low volatility of the data distribution. The average values of the three tests are shown in Figure 2. After tempering at 200–800 °C, the hardness of the 1Cr13 specimens showed a trend of first increasing, then significantly decreasing and finally rapidly increasing with the increase in the tempering temperature, while the hardness of 2Cr13 showed a consistent decreasing trend, after tempering at 200–770 °C, with the increase in the tempering temperature. All impact specimens were subjected to impact testing using a pendulum impact tester with model ZBC2602-B, ZwickRoell, Ulm, Germany. The micromagnetic detection and impact detection positions were set at the center of the V-notch back.

**Figure 3:**
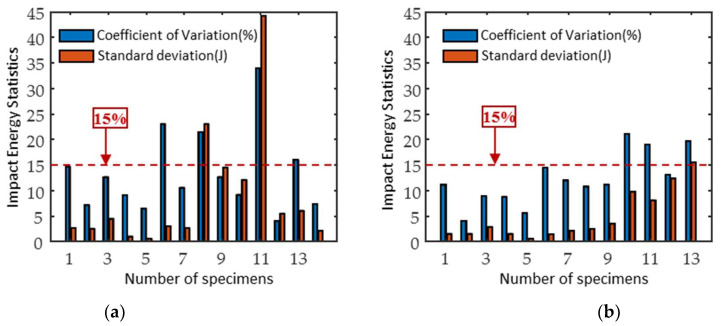
(**a**) 1Cr13 and (**b**) 2Cr13 SD and CV of impact energy.

Figure 2 shows the impact energy of three specimens in the same batch. The trend of the impact energy with the increasing tempering temperature was basically the same for both materials. The impact energy first decreased to the lowest point (caused by temper brittleness) and then increased. The graphical error bar analysis shows that the difference between the three samples in the same batch was relatively large when the impact energy was above 40 J. To quantify the distribution characteristics of the data, the coefficient of variation (CV) and standard deviation (SD) of the impact specimens from the different batches were calculated as shown in Figure 3. The results show that the SD interval of 2Cr13 is narrower than that of 1Cr13, and therefore, its data distribution presents higher stability. Most of the medium and high temperature tempered specimens had an SD greater than 5%, reflecting an increase in the dispersion of the data. When the CV is greater than 15%, it introduces non-negligible systematic errors in the subsequent magnetic signal and impact work predictions.

### 2.2. Micromagnetic Testing

**Figure 4:**
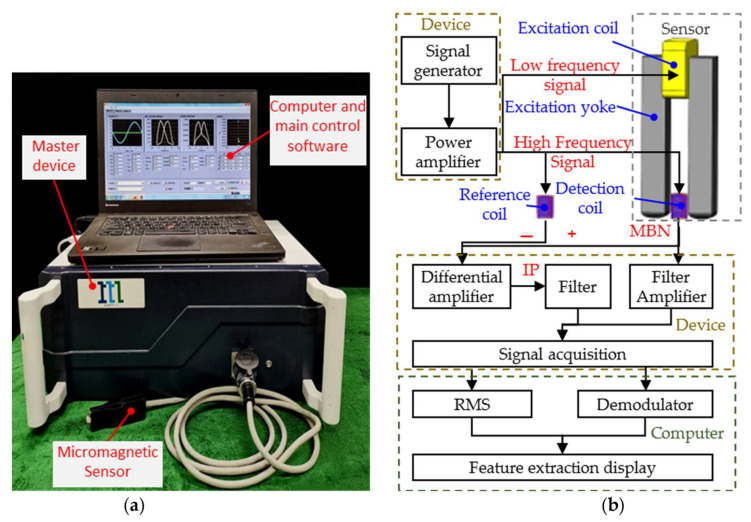
(**a**) The micromagnetic testing system and (**b**) Block diagram of the detection system.

The micromagnetic testing system (Figure 4a) developed by the Research Center for NDT&E at the Beijing University of Technology was employed to carry out the experiments. The system consists of the master device, a micromagnetic sensor, an upper computer and the main control software. The specialized micromagnetic sensor has been developed to accommodate the size of impact specimens. The excitation yoke and the detection element are the two core parts of the sensor. The total height of the excitation yoke was designed to be 40 mm. The yoke leg cross-section size was 6 mm × 6 mm, and was fabricated as an inverted pyramid shape. The mutually inductive, twisted pair detection coils were applied by the detection element. The two wires of the coil were used as excitation and reception signals. The detection coil was encapsulated in the slider and placed between the two yoke legs. During the test, to ensure that the lifting distance was constant, a pre-pressure spring was used to make the wear-resistant layer of the slider close to the surface of the specimen.

**Figure 5:**
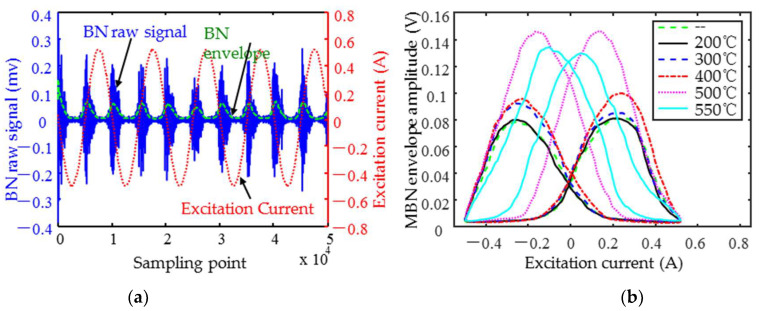
(**a**) Typical waveforms of time-domain signals and (**b**) 0–550 °C tempering MBN butterfly curves for 1Cr13 hardness specimens.

The specialized sensor could detect both MBN and IP signals simultaneously. The detection process is shown in Figure 4b. The multi-channel signal generator generated high and low frequency excitation signals in time division, and then the excitation signals were amplified. The amplified low frequency signal was passed into the excitation coil wound on the yoke. The high frequency excitation coil of the detection coil received the amplified high frequency signal. After the signal was passed in, the specimen was locally magnetized by the alternating magnetic field. For low frequency excitation only, the MBN signal is detected in the receiver of the detector coil. The MBN signal was then filtered and amplified by the conditioning module. The signal was collected by the signal acquisition module. In order to eliminate the effect of mutual inductance during high frequency excitation, the identical reference coil to the detection coil is used and fed with the same high frequency signal. When the specimens were excited by both high and low frequency signals, the differential amplification of the signals received from the detection coil and the reference coil was the IP signal. The filtered IP signal was detected by the acquisition module. The MBN and IP signals were transmitted to the upper computer. Sliding Average and taking envelope measurements were carried out for the MBN signal (Figure 5a). Demodulation was performed on the IP signal. The butterfly curves of MNB (Figure 5b) and IP were plotted and displayed with the series resistance current value of the excitation coil as the horizontal coordinate and the amplitude of the MBN and IP envelopes as the vertical coordinate. The feature parameters extracted from the butterfly curves of MNB and IP are shown in Table 2.

The excitation and sampling parameters, as listed in Table 3, were selected to perform micromagnetic testing on the hardness specimens and the impact specimens of the two materials, respectively. To improve the accuracy of the testing and modeling predictions, three sets of five fixed-point replicate tests were performed per specimen, resulting in 15 data sets for each specimen. For each test, five cycles of MBN and IP signals were acquired, and the typical waveforms are shown in Figure 5a. To eliminate the interference caused by the initial and the remanent magnetization of the material, the middle three magnetization cycles of the MBN signals were intercepted to plot the envelope curves (Figure 5b).

The difference in the heat treatment process caused the shape of the MBN envelope to change as shown in Figure 5b. More specifically, tempering at different temperatures changed microstructures such as carbide precipitation and size, grain size and the number of grain boundaries, as well as the microstructure type and dislocation density, as MBN is sensitive to microstructure changes.

**Figure 6:**
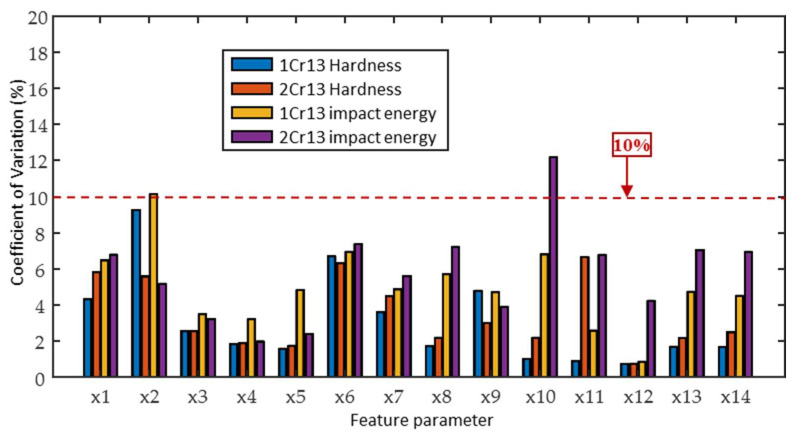
Coefficients of variation of feature parameters.

The results of the quantitative evaluation of the coefficients of variation (CV) of the 14 magnetic parameters are shown in Figure 6. The coefficients of variation were used to characterize the repeatability of the magnetic signal detection system and the stability of the experimental conditions. The analysis results show that, except for x2 and x10, the CV values of the remaining magnetic parameters are controlled below 10%, reflecting the good reproducibility and anti-interference capability of the detection system. To ensure the reliability of the subsequent modeling process, the x2 and x10 signals were excluded from the impact energy prediction modeling.

**Figure 7:**
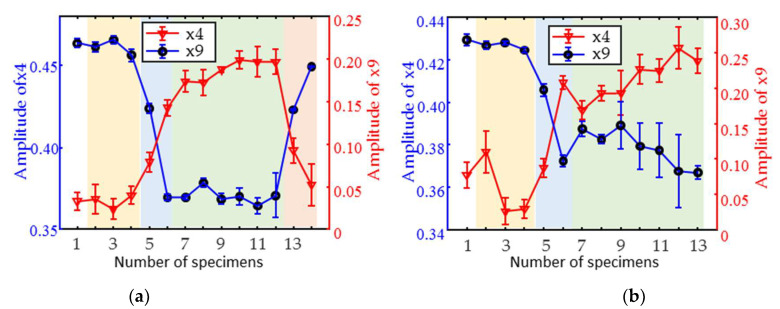
Variations in typical magnetic parameters with tempering temperature of (**a**) 1Cr13 and (**b**) 2Cr13 hardness specimens.

The variation rule for typical magnetic parameters with a tempering temperature for the hardness specimens of the two materials is shown in Figure 7. After 200–400 °C tempering (Number 2~4), the quenched lath martensite transformed into a tempered martensite organization. Due to dislocations, solid solutions and the diffuse strengthening nanoscale alloy carbides leaded to small variations in the magnetic signal. As a result, the magnetic parameters fluctuated in a small range. When tempered at 500–550 °C (Number 5~6), the organization turned into finer tempered martensite; however, the number of alloy carbides increased. Consequently, the magnetic signals varied drastically, and the magnetic parameters rose or fell more sharply.

The organization of both materials changed from tempered martensite to tempered austenite during tempering at 600–750 °C (Number 7~12). As the tempering temperature increased, the organization became more homogeneous, and the dislocations and solid solution strength were weakened. The macroscopic manifestation was the gradual decrease in hardness. As a result, both the magnetic signal and the parameters varied slowly and monotonically. When the tempering temperature of 750 °C (Number 13~14), it exceeded the phase transition temperature of 1Cr13, and austenite appeared in the local organization of the material. The transformation of austenite to martensite upon cooling, coupled with grain refinement and carbide precipitation, resulted in a reversal of the magnetic parameters.

**Figure 8:**
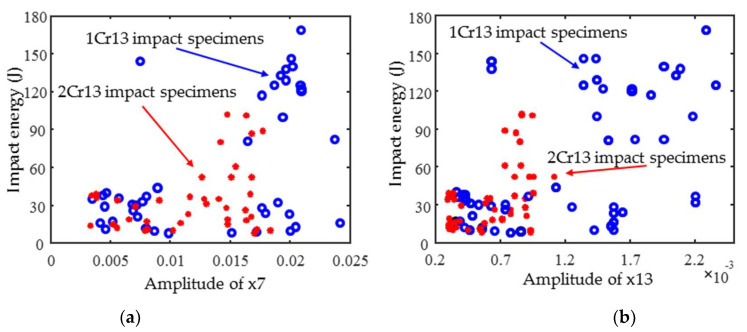
The dependency (**a**) x7 and (**b**) x13 parameters versus impact energy.

The MBN (IP) feature parameter x7 (x13) with the largest mutual information value was selected and plotted against the impact energy as shown in Figure 8. The magnetic features tend to increase with an increasing impact energy. However, there is no obvious linear relationship. A similar phenomenon was observed for the other magnetic features; thus, it is not applicable to correlative coefficient selection and linear regression modeling.

**Figure 9:**
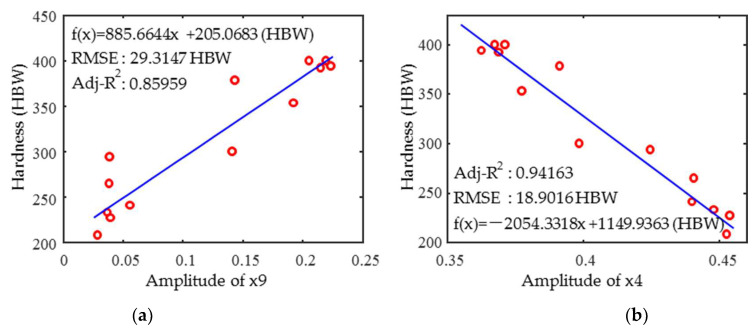
The dependency of 1Cr13 (**a**) x9 and (**b**) x4 parameters versus hardness.

In order to analyze the meaning of the mutual information values of an individual magnetic feature parameter with hardness (impact energy), the relationships were plotted. The parameter with the highest value of mutual information between the magnetic features and the hardness is x9. The apparent linear positive correlation between x9 and hardness is shown in Figure 9a. The mutual information values between other IP feature parameters and the hardness also are greater than 0.7, but there is no significant linear correlation. This is because a mutual information analysis is probability-based and reflects both linear and non-linear correlations. Between the MBN feature parameters, x9 and hardness are linearly and negatively correlated as shown in Figure 9b. The coefficient of determination R^2^ for x4 (0.94) is greater than x9 (0.86), indicating that the mutual information value is not proportional to the coefficient of determination.

**Figure 10:**
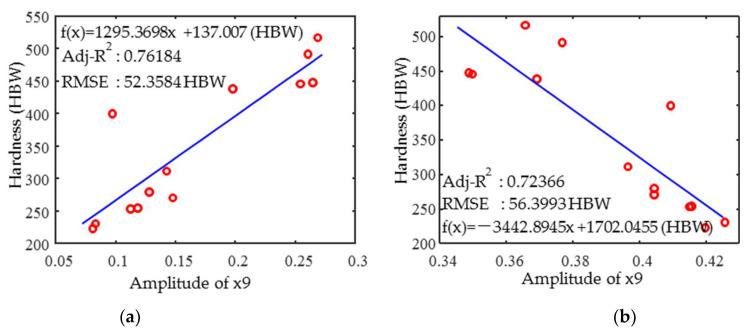
The dependency of 2Cr13 (**a**) x9 and (**b**) x4 parameters versus hardness.

As shown in Figure 10, the relationship between x9 (x4) and the hardness of 2Cr13 is similar to that of 1Cr13, with a linear positive (negative) correlation, respectively, but with a smaller determination coefficient than that of the 1Cr13 material.

## 3. Modeling

**Figure 11:**
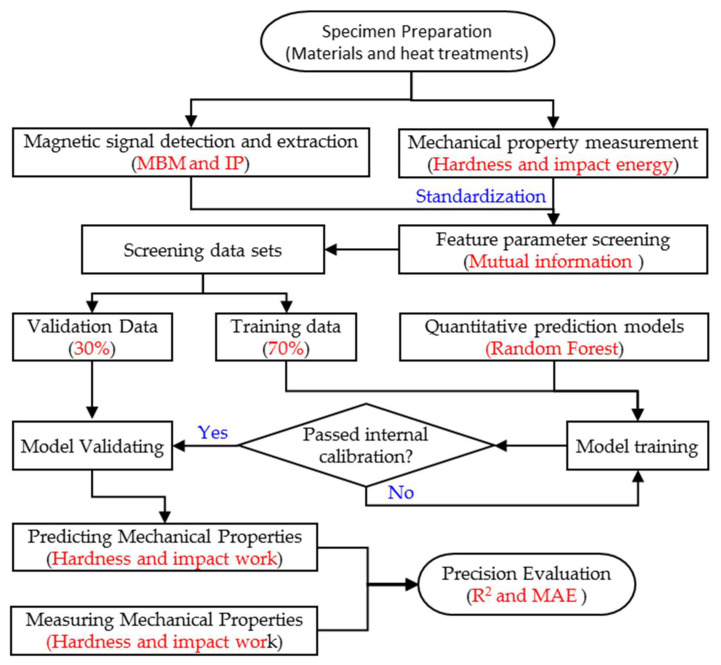
Flow chart of the quantitative prediction.

Since the physical mechanism between micromagnetism and mechanical properties has not been clearly revealed, a micromagnetic testing method characterizes the relationship between magnetic feature parameters and mechanical property indexes through calibrated experiments. The above results indicate a significant non-linear correlation between the magnetic parameters and mechanical properties. To achieve a highly accurate quantitative prediction of mechanical properties, an intelligent algorithm combining mutual information parameter screening and the random forest modeling method has been proposed for model construction. The specific implementation process of this technical solution is shown in Figure 11. Specimens with different mechanical properties were first prepared. Magnetic signals were collected, and the feature parameters were extracted using a micromagnetic detecting system. Mechanical properties were tested by the destructive method. The two sets of raw data were standardised (i.e., the mean was subtracted and divided by the standard deviation). The mutual information value between the magnetic properties and the mechanical properties was calculated from the standardised data. The sensitive magnetic parameters with a strong correlation (including both linear and non-linear) with the mechanical properties were screened out by setting the mutual information threshold (≥0.6). To construct a training set for training the random forest regression model, a stratified sampling strategy was used to randomly select 70% of the paired magnetic feature-mechanical property data from the screened data set. The remaining 30% of the data were used as an independent validation set to systematically evaluate the model’s prediction accuracy by calculating the dual metrics of the coefficient of determination (R^2^) and the mean absolute error (MAE). This process achieves a quantitative analysis from feature selection to model validation, ensuring that the prediction model has both feature sensitivity and generalisability.

### 3.1. Feature Parameters Screening

For an RFM with given structural parameters, the dimensionality and sensitivity of the input features affect the prediction accuracy of hardness (impact energy). Model accuracy and computational efficiency would be reduced by incorporating features with a low target relevance into the model. Mutual Information (MI) is the classical measure in information theory. Mutual information is the independent probabilistic relationship between features and categories, including linear and non-linear correlations. By calculating the mutual information values of the magnetic feature parameters and the mechanical property indexes, it is possible to remove some features that are not relevant or redundant to the hardness (impact energy), thus reducing the dimensionality of the feature parameters input to the random forest model.

The mutual information value is determined by the probability of simultaneous and separate occurrences of the magnetic feature parameter and the mechanical property index, and it is able to evaluate the dependence of the two. A dependence of the two variables is indicated by a higher mutual information value. It means that the two features are independent of each other if the mutual information value is zero. The equation for calculating the mutual information value is as follows:

(1) $$I\left(t,c\right)=\log\frac{p\left(t,c\right)}{p(t)\times p(c)}$$

where *t* represents the features (in this paper, the magnetic feature parameters); *c* represents the categories (in this paper, the mechanical properties index); $p\left(t,c\right)$ represents the probability of the *t* and *c* occurring simultaneously; $p\left(t\right)$ represents the probability of t occurring alone; and $p\left(c\right)$ represents the probability of c occurring alone.

The magnetic feature parameters and hardness (impact energy) in this paper were *t* and *c* in the above equation, respectively. When calculating the mutual information value of t and *c*, the probabilities can be approximated by the frequency of corresponding occurrences in the data set. Assuming that the frequency of the simultaneous occurrence of *t* and *c* is *A*, *N* represents the total amount of data in the data set, while *B* and *C* represent the number of times *t* and *c* occur in the data set, respectively. The mutual information value is approximated as follows:

(2) $$I\left(t,c\right)=\log\frac{A\times N}{B\times C}$$

To quantitatively assess the relevance of individual magnetic parameters for hardness (impact work), the mutual information values between them were calculated.

### 3.2. Random Forest Modeling

**Figure 12:**
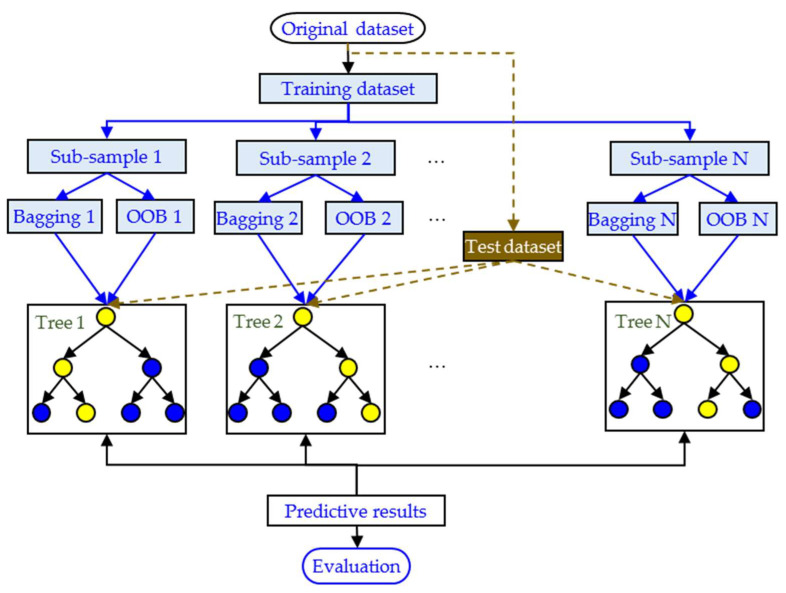
Flow chart for random forest algorithm.

Random forest is a machine learning algorithm based on integrated learning, which improves model performance by constructing multiple decision trees and synthesising their predictions. The algorithm uses a double random mechanism (sample sampling and feature sampling) to train each subtree, which is effective at avoiding the risk of overfitting and particularly good at resolving complex non-linear mapping relationships in small sample scenarios. The random forest prediction process is shown in Figure 12. The original data set is first divided into a training set and a validation set. For training, multiple subsample sets (bagging) are generated from the training set using put-back randomly drawn samples. The unsampled samples constitute the out-of-bag (OOB) data. A regression decision tree is trained for each subsample set, and some features are randomly selected for optimal segmentation during node splitting. The splitting threshold is determined by minimising the mean square error (MSE). Hundreds of decision trees are repeatedly constructed to form a ‘forest’. Each tree independently learns the relationship between the magnetic features and mechanical properties mapping. The generalisation performance of the model is evaluated in real time by out-of-bag (OOB) error to avoid overfitting. For a prediction, a test data set is fed into all decision trees. The average of the predicted values from each tree is taken as the final result. The coefficient of determination (R^2^) and root mean square error (RMSE) are calculated to evaluate prediction accuracy. The process effectively captures complex non-linear relationships through a dual stochastic mechanism (sample + feature) and integrated learning, and is particularly suited to small-sample, high-dimension data scenarios in the field of micromagnetic detection.

## 4. Results and Discussion

**Figure 13:**
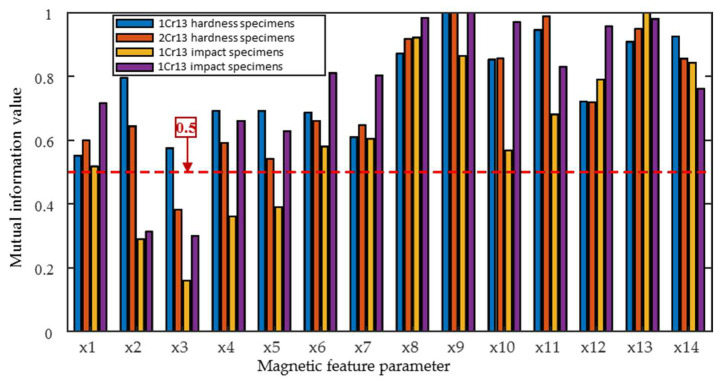
Mutual information values between magnetic feature parameters and hardness and impact energy.

Figure 13 shows the analysis results of the mutual information between all fourteen magnetic parameters and hardness (impact energy). In general, the mutual information value of IP is greater than that of MBN, and the value of the impact specimens is less than that of the hardness specimens due to the measurement error of impact energy.

**Figure 14:**
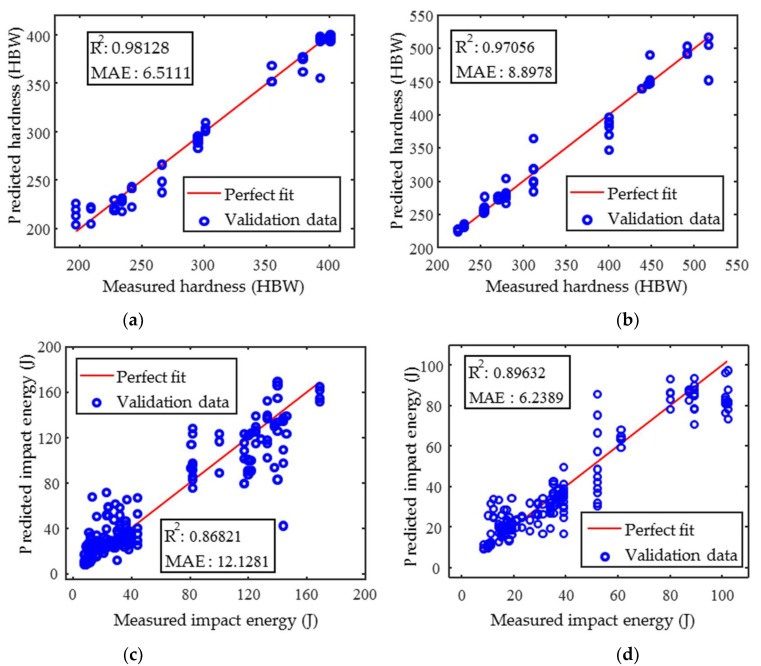
Random forest quantitative prediction results (**a**) hardness of 1Cr13, (**b**) hardness of 2Cr13, (**c**) impact energy of 1Cr13 and (**d**) impact energy of 2Cr13.

Following the above procedure, the predicted results of hardness and impact energy can be obtained as shown in Figure 14. There are differences in the prediction accuracy of the random forest model for hardness and impact energy. The coefficient of determination (R^2^) of the linear fit between the predicted and measured hardness values was greater than 0.97, and the accuracy of the hardness prediction was higher than that of the impact energy.

The hardness data for 2Cr13 are poorly homogenized (the data are concentrated in two data segments) and the amount of data is relatively small (a small number of specimens) compared to 1Cr13. Therefore, the prediction accuracy of the 2Cr13 random forest model is lower than that of 1Cr13. The mean absolute errors (MAEs) of the hardness predictions for 1Cr13 and 2Cr13 were less than 7 HBW (Figure 14a) and 9 HBW (Figure 14b), respectively.

When the measured value of the impact energy is greater than 40 J, the difference between the impact energy measurements of the three parallel specimens shown in Figure 2 is significant. The measurement error of the impact energy caused the predictive accuracy of the model decreases, and the R^2^ is less than 0.9 for both materials. The measurement error of the impact energy for 1Cr13 is greater than that of 2Cr13; therefore, its predictive accuracy is also less. The MAE of the predictions for 1Cr13 and 2Cr13 are 12.13 J (Figure 14c) and 6.24 J (Figure 14d), respectively. The random forest model prediction method significantly improves the prediction accuracy of hardness and impact energy compared the single factor characterization method. The random forest model is relatively accurate in predicting hardness and impact energy for both materials and can be used to evaluate the hardness and impact energy of specimens subjected to different heat treatments.

## 5. Conclusions

In this study, the magnetic feature parameters (derived from MBN and IP signals) and target mechanical properties (surface hardness and impact energy) of two martensitic stainless steels (1Cr13 and 2Cr13) were experimentally characterised. A hybrid predictive methodology combining mutual information (MI)-based parameter screening with random forest (RF) modelling was developed to achieve accurate quantitative predictions.

Methodologically, the MI values between the magnetic parameters and hardness/impact energy were first calculated to identify the features with strong linear and non-linear correlations. By eliminating redundant features, the input dimensionality has been reduced, improving prediction accuracy and computational efficiency. This approach overcomes the limitations of conventional feature selection methods, which often overlook non-linear dependencies. A random forest prediction model (RFM) was implemented to handle the complex relationships between magnetic features and mechanical properties. Compared to traditional multiple linear regression, the RFM demonstrated a superior ability to resolve non-monotonic relationships. In addition, the RFM outperformed BP neural networks in small sample scenarios, achieving higher prediction stability and generalisation.

The quantitative results showed that the mean absolute error (MAE) for predicting surface hardness was reduced to less than 9 HBW for both steel grades. For the impact energy predictions, MAE values of 12.13 J and 6.24 J were achieved for 1Cr13 and 2Cr13, respectively. These results highlight the adaptability of the model to material-specific variations.

Beyond specific applications, this MI-RF framework advances the NDE of martensitic steels and provides a versatile mechanical property prediction paradigm. By integrating a feature-level dependency analysis with ensemble learning, this study addresses material characterisation challenges with limited data and opens new avenues for future magnetic materials assessment research.

## Figures and Tables

**Table 1:** Chemical composition of tested materials.

	Elements	C	Mn	Si	P	Cr	Mo	Ni	V	Cu	S
Alloy											
1Cr13		0.133	0.487	0.352	0.020	12.00	0.029	0.488	0.050	0.043	<0.005
2Cr13		0.189	0.504	0.330	0.023	13.18	0.041	0.384	0.060	0.058	<0.005

**Table 2:** Feature parameters of MBN and IP signals.

Signal Type	Indicators	Descriptions
Barkhausen Noise	x1	Peak height of the MBN butterfly curve
	x2	Peak position of the MBN butterfly curve
	x3	Full width at 75% of maxima of the MBN butterfly curve
	x4	Full width at half maxima of the MBN butterfly curve
	x5	Full width at 25% of maxima of the MBN butterfly curve
	x6	Intercept of the MBN envelope at the vertical axis
	x7	Mean value of the MBN envelope for a single magnetization cycle
Incremental magnetic permeability	x8	Peak height of the IP butterfly curve
	x9	Peak position of the IP butterfly curve
	x10	Full width at 75% of maxima of the IP butterfly curve
	x11	Full width at half maxima of the IP butterfly curve
	x12	Full width at 25% of maxima of the IP butterfly curve
	x13	Intercept of the IP envelope at the vertical axis
	x14	Mean value of the IP envelope for a single magnetization cycle

**Table 3:** Experimental system parameter setting.

Operational conditions	Properties	Units
Low frequency excitation	Frequency	200 Hz
	Amplitude	1 App
	Cycle	5
High Frequency excitation	Frequency	100 kHz
	Amplitude	0.5 Vpp
Sampling	Rate	2 MS/s
	Point	50,000

## Data Availability

The data presented in this study are available on request from the corresponding author.
